# Fate tracking reveals differences between Reelin^+^ hepatic stellate cells (HSCs) and Desmin^+^ HSCs in activation, migration and proliferation

**DOI:** 10.1111/cpr.13500

**Published:** 2023-05-28

**Authors:** Ning Chen, Shenghui Liu, Dan Qin, Dian Guan, Yaqing Chen, Chenjiao Hou, Songyun Zheng, Liqiang Wang, Xiangmei Chen, Wei Chen, Lisheng Zhang

**Affiliations:** ^1^ College of Veterinary Medicine/Bio‐medical Center/ Huazhong Agricultural University Wuhan China; ^2^ College of Life Science and Technology Huazhong Agricultural University Wuhan China; ^3^ Department of Nephrology, Chinese PLA General Hospital, Chinese PLA Institute of Nephrology, State Key Laboratory of Kidney Diseases National Clinical Research Center for Kidney Diseases Beijing China; ^4^ Department of Food Science and Nutrition, College of Biosystems Engineering and Food Science Zhejiang University Hangzhou China

## Abstract

The activation of hepatic stellate cells (HSCs) is the main cause of liver fibrogenesis in response to different etiologies of chronic liver injuries. HSCs are heterogeneous, but the lack of specific markers to distinguish different HSC subset hinders the development of targeted therapy for liver fibrosis. In this study, we aim to reveal new HSC subsets by cell fate tracking. We constructed a novel ReelinCreERT2 transgenic mouse model to track the fate of cells expressing Reelin and their progeny (Reelin^+^ cells). And we investigated the property of Reelin^+^ cells, such as differentiation and proliferation, in hepatotoxic (carbon tetrachloride; CCl_4_) or cholestatic (bile duct ligation; BDL) liver injury models by immunohistochemistry. Our study revealed that Reelin^+^ cells were a new HSC subset. In terms of activation, migration, and proliferation, Reelin^+^ HSCs displayed different properties from Desmin^+^ HSCs (total HSCs) in cholestatic liver injury model but shared similar properties to total HSCs in hepatotoxic liver injury model. Besides, we did not find evidence that Reelin^+^ HSCs transdifferentiated into hepatocytes or cholangiocytes through mesenchymal‐epithelial transition (MET). In this study, our genetic cell fate tracking data reveal that ReelinCreERT2‐labelled cells are a new HSC subset, which provides new insights into targeted therapy for liver fibrosis.

## Graphical abstract

- Cell lineage tracing reveals that HSCs are marked by ReelinCreERT2, and Reelin is a new marker of an HSC subset.
- ReelinCreERT2‐labled HSCs show different properties in BDL‐induced fibrotic livers and similar characteristics in CCl_4_‐induced fibrotic livers compared to Desmin^+^ HSCs.
- In cholestatic model, Desmin^+^Reelin^−^ HSCs accumulate around the portal triad with a significant activation and proliferation activity, but Desmin^+^ Reelin^+^ HSCs do not accumulate around the portal triad or proliferate, and only a small part is activated.
- In hepatotoxic model, Desmin^+^Reelin^+^ HSCs and Desmin^+^Reelin^−^ HSCs both accumulated around the central vein with remarkable activation, and proliferation.
- Desmin^+^Reelin^+^ HSCs do not transdifferentiate into hepatocytes or cholangiocytes through MET in cholestatic or hepatotoxic model.

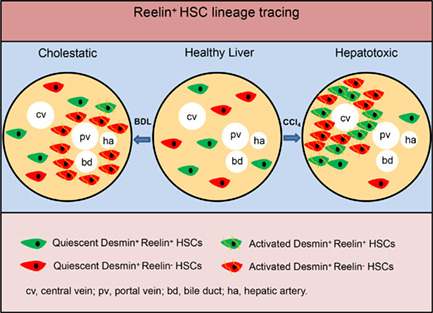

The differences between Reelin^+^ HSCs and Desmin^+^ HSCs.

## 1 INTRODUCTION

Reelin is a secreted extracellular glycoprotein with a molecular weight of 420 kDa that plays a vital role in neuronal migration, dendritic spine formation and synaptogenesis.^1^, ^2^ Abnormal expression of Reelin has been associated with various neuropsychiatric disorders, such as schizophrenia, epilepsy and Alzheimer's disease.^3^, ^4^, ^5^ Although Reelin is primarily known for its role in brain, accumulating evidence indicates its involvement in liver fibrosis. Specifically, Reelin have been found to be up‐regulated in patients with liver cirrhosis in the liver and plasma.^6^ In addition, research has shown that blood Reelin levels are significantly elevated in patients with liver fibrosis or cirrhosis, and patients with hepatocellular carcinoma (HCC) have markedly higher concentrations of Reelin compared to patients with liver cirrhosis.^7^ Furthermore, a separate study suggests that Reelin expression in the liver is positively associated with the stage of liver fibrosis.^8^ Besides these, one study reports the expression of Reelin in hepatocytes,^6^ while other studies have shown that Reelin is expressed in both hepatocytes and hepatic stellate cells (HSCs).^9^, ^10^ And others demonstrate that Reelin is only expressed in HSCs.^8^, ^11^, ^12^ Furthermore, some studies report that Reelin is detected in hepatoblasts.^13^ Thus, there exists a huge debate over which type of cells express Reelin and the role of Reelin in liver fibrogenesis needs to be further investigated.

HSCs are mesenchymal cells and exhibit fibroblast and pericyte characteristics.^14^, ^15^, ^16^ Following liver injury, quiescent HSCs are activated and differentiate into migratory, contractile and proliferative myofibroblasts (MFs) to secrete extracellular matrix (ECM).^15^, ^17^, ^18^ Genetic cell lineage tracking has shown that HSCs are the primary source of MFs^19^, ^20^, ^21^ and the activation of HSCs is the main cause of liver fibrogenesis.^22^, ^23^, ^24^ And some lineage tracking studies have demonstrated that HSCs differentiate into hepatocytes and cholangiocytes through mesenchymal‐to‐epithelial transition (MET),^25^, ^26^, ^27^ but others have revealed no HSCs undergo MET.^19^, ^28^, ^29^ So far, whether HSCs undergo MET is still a scientific question to be addressed. Although HSCs play a major role in response to various types of liver fibrosis, the fibrogenic phenotype and mechanisms are different.^30^, ^31^, ^32^, ^33^ Bile duct ligation (BDL)‐ and carbon tetrachloride (CCl_4_)‐induced liver injuries are two distinct liver fibrosis models that mimic cholestasis and hepatotoxicity, respectively.^33^, ^34^, ^35^ In addition, single‐cell RNA sequencing reveals that HSCs are heterogeneous,^12^, ^13^, ^36^ but lack of specific markers to distinguish various HSC subsets makes it difficult to targeted treatment of liver fibrosis.^37^, ^38^, ^39^, ^40^

In this study, our genetic cell fate tracking data revealed that ReelinCreERT2‐labelled HSCs displayed different properties from Desmin^+^ HSCs (total HSCs) in BDL‐induced fibrotic livers and similar properties to Desmin^+^ HSCs in CCl_4_‐induced fibrotic livers.

## 2 MATERIALS AND METHODS

### 2.1 Mice

The animals in this study were against a C57BL6/J background. Rosa26mTmG reporter mice were obtained from Jackson Laboratory. ReelinCreERT2 mice were constructed by Biocytogen (Beijing, China). The P2A‐iCreERT2 cassette was inserted after the stop codon TGA of Exon64 of Reelin and the knock‐in mice were prepared based on the CRISPR/Cas9‐based system developed by Biocytogen. ReelinCreERT2 mice were crossed with Rosa26mTmG reporter mice to generate Reelin^CreERT2^; Rosa26mTmG^flox^ (R26T/G^f^) mice used for subsequent experiments. ReelinCreERT2 genotype identification was performed by using forward primer 5′‐CTCTGCTGCCTCCTGGCTTCT and reverse primer 5′‐TCAATGGGCGGGGGTCGTT. Rosa26mTmG reporter mice genotype identification was conducted by using forward primer 5′‐ TATTCTGTCCCTAGGCGGTGAAGTCT and reverse primer 5′‐ CCTGTCCCTGAACATGTCCATCAG. The schematic diagram of the target carrier of ReelinCreERT2 is as follows:
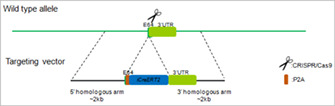


### 2.2 Tamoxifen injection and fibrosis induction

Mice were maintained under specific pathogen‐free conditions at the animal facilities of Huazhong Agricultural University and used for fibrosis at ages 8–12 weeks random. ReelinCreERT2 activity was induced by intraperitoneal injections of tamoxifen (TAM) (Sigma, T5648, Missouri, USA) at the dose of 100 mg/kg on a daily basis for 3 days starting 7 days before treatment. TAM was dissolved in corn oil (Aladdin, Shanghai, C116023) at the dose of 20 mg/mL. Reelin^CreERT2^; Rosa26mTmG^flox^ mice treated only with TAM or corn oil were five per group. For hepatotoxic liver injury, Reelin^CreERT2^; Rosa26mTmG^flox^ mice (*n* = 5) were treated with intraperitoneal injections of CCl_4_ (Aladdin, C112043, China) at the dose of 1 mL/kg body weight, two times a week for 6‐week. CCl_4_ was dissolved in corn oil at a ratio of 1:4. The control group (*n* = 5) were treated with intraperitoneal injections of coin oil. Mice treated with CCl_4_ or corn oil were sacrificed 48 h after the last injection. For cholestatic liver injury, Reelin^CreERT2^; Rosa26mTmG^flox^ mice (*n* = 5) common bile duct was ligated twice with 6–0 silk sutures, and the sham group (*n* = 5) was operated similarly, except that the bile duct was not ligated. Mice were sacrificed 2 weeks after surgery. Bromodeoxyuridine (BrdU) (Sigma, B5002) was dissolved in saline and treated with intraperitoneal injection at the dose of 50 mg/kg body weight every 2 h for 4 times, the last injection was taken 24 h before mice were sacrificed. Three WT mice were assigned to each of the following groups: sham‐operated, BDL‐operated, control and CCl_4_‐treated. All procedures followed the Huazhong Agricultural University Guidelines for the Care and Use of Laboratory Animals.

### 2.3 Immunofluorescent assay

Samples were fixed in 4% paraformaldehyde (PFA), embedded in paraffin, cut into 4 μm sections, dewaxed, hydrated and subsequently incubated with antibodies. Fluorescence was bleached with 3% H_2_O_2_ in methanol for 15 min. For antigen retrieval, samples were heated in 10 mM sodium citrate buffer (pH 6.0) for 20 min. Sections were blocked with 10% goat serum for 30 min and incubated with primary antibodies, anti‐BrdU (Servicebio, GB12051, Wuhan, China), anti‐CD68 (Servicebio, GB113109), anti‐CK19 (Servicebio, GB12197), anti‐Col1a1 (Servicebio, GB11022‐3), anti‐Desmin (Servicebio, GB12081), anti‐EpCAM (Servicebio, GB1127), anti‐GFP (Proteintech, 50,430‐AP, Wuhan, China), anti‐GFP (Santa Cruz, sc‐9996, Texas, USA), anti‐GS (glutamine synthetase) (Santa Cruz, sc‐74430), anti‐HNF4α (Abcam, Ab41898, Cambridgeshire, UK), anti‐Ki67 (Invitrogen, PA5‐19462, Massachusetts, USA), anti‐Reelin (Santa Cruz, sc‐25346), anti‐tdTomato (MBL, PM005, Tokyo, Japan), anti‐Vimentin (Abcam, Ab92547), and anti‐α‐SMA (Servicebio, GB111364). Subsequently, sections were incubated with fluorophore‐conjugated secondary antibodies (2.5 μg/mL, Invitrogen, A‐11034, A‐21424), nuclei co‐staining with 4, 6‐diamidino‐2‐phenylindole (DAPI) (Abcam, ab104139). Images were acquired with a laser scanning confocal microscope (Carl Zeiss Microscopy, LSM710, Jena, Germany), and were analysed by Zen software with fixed parameters.

### 2.4 Histology analysis

Liver tissues were immobilized with 4% PFA, dehydrated, embedded in paraffin, sectioned at 4 μm and processed for Sirius red staining (Solarbio, G1472, Beijing, China). All steps are according to manufacturer's instructions.

### 2.5 Software‐intensity measurement

Image Pro Plus (Image Pro Plus v.7: Media Cybernetics; Bethesda, MD), as an analysis program, was used to analyse and quantify data from photomicrographs.

### 2.6 Statistical analysis

Statistical analyses were performed using the GraphPad Prism 6 (GraphPad). Data are expressed as means ± SEM. Comparisons between two groups were performed using the unpaired two‐tailed Student's *t*‐test. Statistical significance was presented at the level of *p* > 0.05 (no significance, ns), *p < 0.05, **p < 0.01, ***p < 0.001.

## 3 RESULTS

### 3.1 ReelinCreERT2 labels HSCs in mouse livers

**FIGURE 1:**
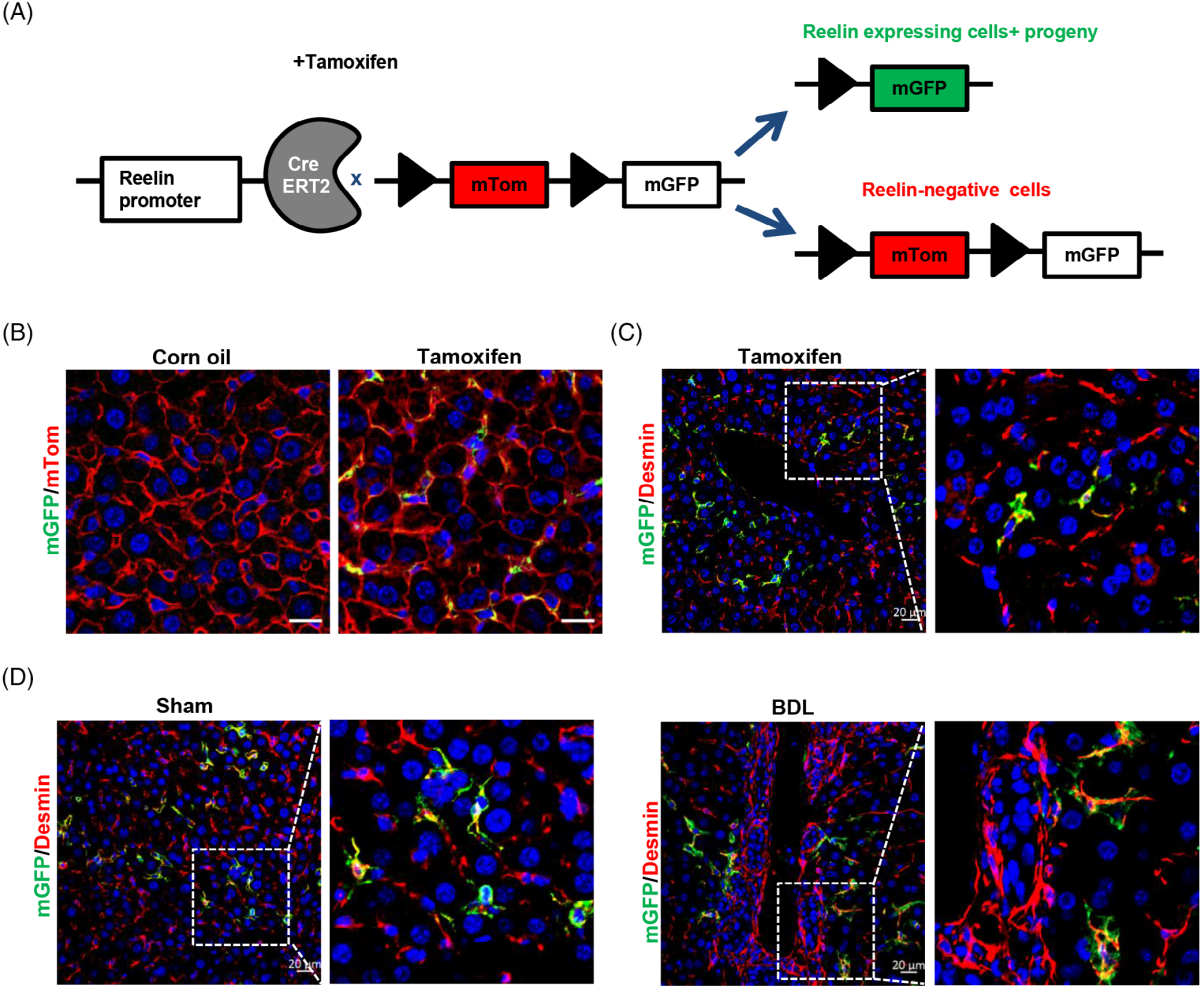
Reelin is expressed in hepatic stellate cells (HSCs) in mouse livers. (A) Schematic diagram showing membrane‐tagged tomato fluorescence protein (mTom)/membrane‐tagged green fluorescence protein (mGFP) reporter gene expression in the absence and presence of tamoxifen (TAM)‐inducible CreERT2‐mediated recombination. (B) mGFP/mTom double staining indicated that mGFP was induced after treated with TAM in Reelin^CreERT2^; R26T/G^f^ mouse livers. (C) mGFP/Desmin double staining indicated that mGFP was expressed in part of HSCs in normal mouse livers. (D) mGFP/Desmin double staining demonstrated that Desmin^+^ HSCs gathered in bile duct ligation (BDL)‐induced fibrotic livers, but mGFP^+^ HSCs did not. Scale bar in B represents 50 μm. Scale bar in C and D represents 20 μm.

In order to track and explore the role of Reelin‐expressing cells and their progeny in mouse livers, we constructed Reelin^CreERT2^; Rosa26mTmG^flox^ (R26T/G^f^) mouse model. In this model, after TAM treatment, tomato sequence was excised by Cre and membrane‐tagged green fluorescence protein (mGFP) started to be expressed (Figure 1A). To verify this model is available, we treated Reelin^CreERT2^; R26T/G^f^ mice with TAM or corn oil. And we observed that ReelinCreERT2‐marked cells expressed mGFP only in the presence of TAM (Figure 1B) in the liver, which suggested that Reelin^CreERT2^; R26T/G^f^ mouse was a credible model to label Reelin‐expressing cells and their progeny. Next, we explored the type of ReelinCreERT2‐labelled cells in mouse livers. mGFP/Desmin double staining showed that some Desmin^+^ HSCs (total HSCs) were mGFP^+^ (Figure 1C), which indicated that ReelinCreERT2 labels cells are HSCs. We also observed no hepatocytes or hepatoblasts expressed mGFP, which indicated that ReelinCreERT2‐labed cells are not hepatocytes or hepatoblasts (Figure S1A). As only some HSCs express mGFP and HSCs are the main cause of liver fibrosis, we want to know the difference between Desmin^+^ HSCs and mGFP^+^ HSCs (Reelin‐expressing HSCs and their progeny) in fibrotic livers. After BDL operation, Sirius red and immunofluorescent staining indicated that collagen fibre, alpha‐smooth muscle actin (α‐SMA) and collagen type I alpha 1 (Col1a1) expression significantly increased (Figure S1B,C), indicating that mouse livers developed significant fibrosis. Immunohistochemistry staining of mGFP and Desmin indicated that Desmin^+^ HSCs gathered in BDL‐induced fibrotic mouse livers, but mGFP^+^ HSCs did not (Figure 1D).

### 3.2 ReelinCreERT2‐labelled HSCs do not accumulate around the portal vein and only a small fraction is activated in BDL‐induced fibrotic livers

**FIGURE 2:**
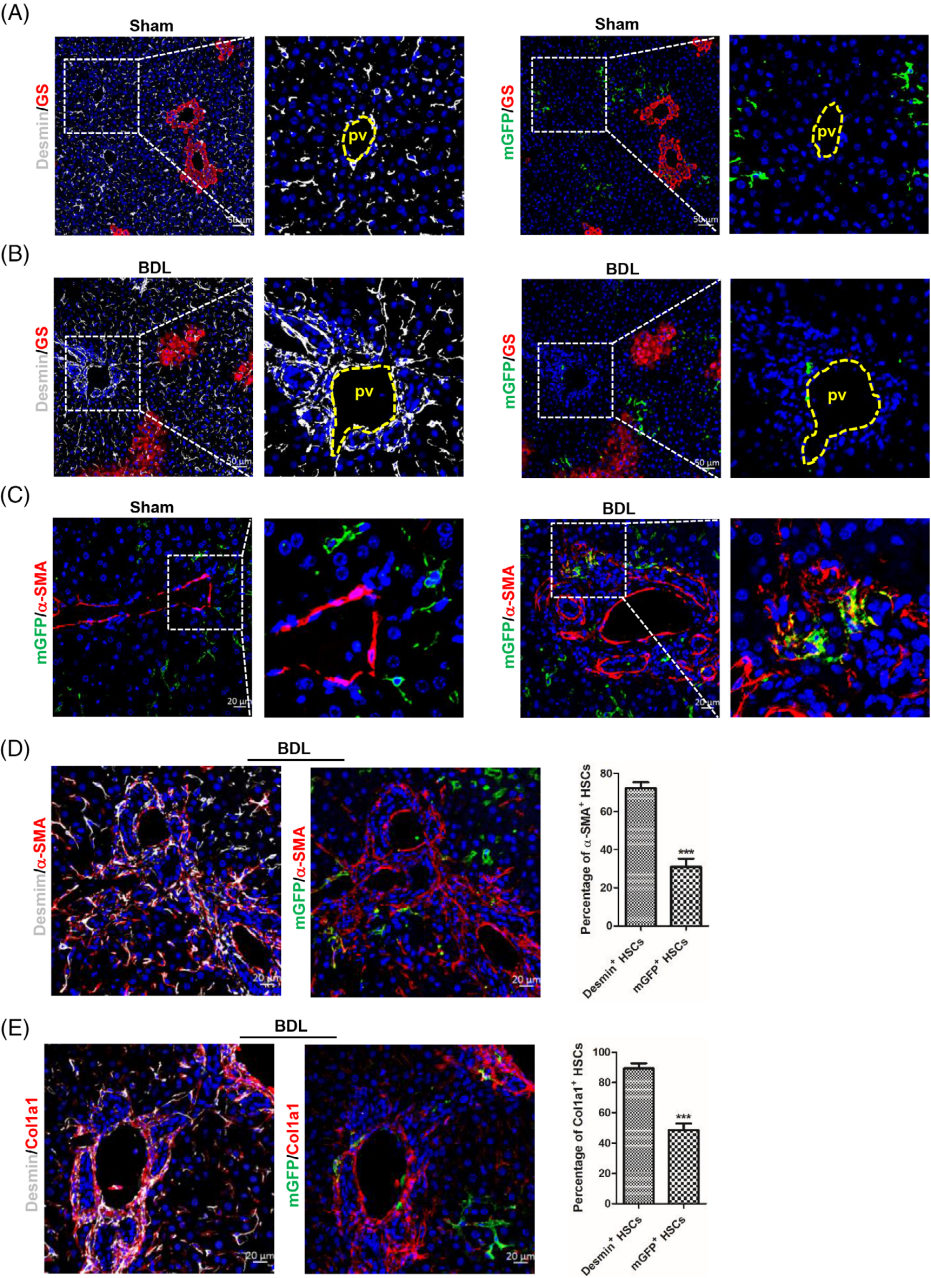
ReelinCreERT2‐labelled hepatic stellate cells (HSCs) do not accumulate around the portal area and fewer are activated compared to Desmin^+^ HSCs in bile duct ligation (BDL)‐induced fibrotic livers. (A) Desmin/glutamine synthetase (GS) or membrane‐tagged green fluorescence protein (mGFP)/GS double staining in serial sections determined that mGFP^+^ HSCs and Desmin^+^ HSCs were scattered throughout the parenchyma in sham‐operated Reelin^CreERT2^; R26T/G^f^ mouse livers. (B) Desmin/GS or mGFP/GS double staining in serial sections in BDL‐operated Reelin^CreERT2^; R26T/G^f^ mouse livers indicated that Desmin^+^ HSCs accumulated around the portal vein (pv), whereas mGFP^+^ HSCs were scattered throughout the parenchyma. (C) mGFP/α‐SMA double staining showed that mGFP^+^ HSCs were activated in BDL‐induced Reelin^CreERT2^; R26T/G^f^ mouse fibrotic livers. (D) Desmin/α‐SMA or mGFP/α‐SMA double staining in serial sections showed that fewer mGFP^+^ HSCs expressing α‐SMA compared to Desmin^+^ HSCs in BDL‐induced Reelin^CreERT2^; R26T/G^f^ mouse fibrotic livers. (E) Desmin/ Col1a1 or mGFP/Col1a1 double staining in serial sections determined that fewer mGFP^+^ HSCs expressing Col1a1 compared to Desmin^+^ HSCs in BDL‐induced Reelin^CreERT2^; R26T/G^f^ mouse fibrotic livers; *n* = 5 per group. Data are reported as means ± SEM. Comparisons between two groups were performed using the unpaired two‐tailed Student's *t*‐test. Statistical significance was presented at the level of *p* > 0.05 (ns), *p < 0.05, **p < 0.01, ***p < 0.001. Scale bar in A and B represents 50 μm. Scale bar in C, D and E represents 20 μm.

The above finding revealed that in BDL‐induced fibrotic mouse livers Desmin^+^ HSCs gathered, but mGFP^+^ HSCs did not (Figure 1D). So, we investigated whether there were differences between Desmin^+^ HSCs and mGFP^+^ HSCs in migration. Immunohistochemistry staining of mGFP, Desmin, and GS (a marker of central vein is not a marker of portal vein) in serial sections showed that both mGFP^+^ and Desmin^+^ HSCs were scattered throughout the parenchyma in sham‐operated livers (Figure 2A). Whereas Desmin^+^ HSCs accumulated around the portal vein in BDL‐induced fibrotic livers, mGFP^+^ HSCs were still scattered throughout the parenchyma (Figure 2B). These findings suggested that significant differences existed in migration between Desmin^+^ and mGFP^+^ HSCs in BDL‐induced fibrotic livers. Studies reported that quiescent HSCs were activated to MFs in injured livers.^41^ We explored the activation ability of mGFP^+^ HSCs. Immunostaining of mGFP and α‐SMA indicated that mGFP^+^ HSCs were able to activate to MFs in BDL‐induced fibrotic livers (Figure 2C). As only part of Desmin^+^ HSCs are mGFP^+^, and Desmin/α‐SMA and mGFP/α‐SMA double staining in serial sections showed that 72.35% Desmin^+^ HSCs expressed α‐SMA, but only 31.01% mGFP^+^ HSCs expressed α‐SMA in BDL‐induced fibrotic livers (Figure 2D), which suggested that fewer mGFP^+^ HSCs were activated than Desmin^+^ HSCs. To confirm the finding that fewer mGFP^+^ HSCs were activated in BDL‐induced fibrotic livers, we analysed Desmin/Col1a1 and mGFP/Col1a1 immunostaining in serial sections and got a similar result, which showed that 48.53% mGFP^+^ HSCs and 89.34% Desmin^+^ HSCs expressed Col1a1 (Figure 2E).

### 3.3 ReelinCreERT2‐labelled HSCs do not proliferate in BDL‐induced fibrotic livers

**FIGURE 3:**
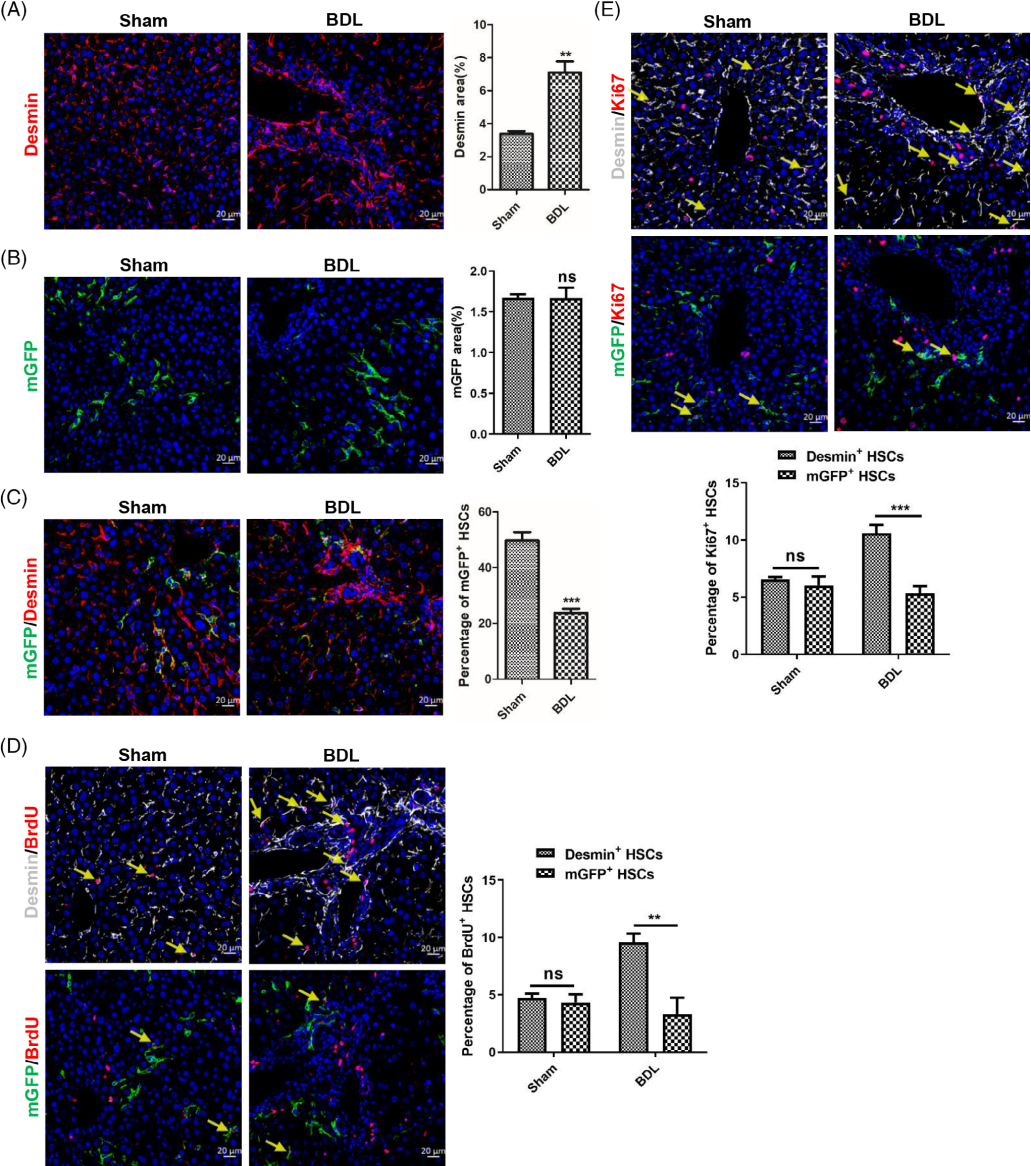
The proliferation capacity of ReelinCreERT2‐labelled hepatic stellate cells (HSCs) has no significant differences between sham‐operated and bile duct ligation (BDL)‐induced fibrotic livers. (A) Desmin immunofluorescent staining indicated that the number of Desmin^+^ HSCs increased significantly in BDL‐induced injured livers. (B) membrane‐tagged green fluorescence protein (mGFP) immunofluorescent staining indicated that the number of mGFP^+^ HSCs did not increase in BDL‐induced injured livers. (C) mGFP/Desmin double staining indicated that the percentage of mGFP^+^ HSCs accounted for Desmin^+^ HSCs significantly reduced in BDL‐induced fibrotic livers. (D) Desmin/bromodeoxyuridine (BrdU) or mGFP/BrdU double staining in serial sections demonstrated that Desmin^+^ HSCs proliferative properties significantly increased in BDL‐induced fibrotic liver, but mGFP^+^ HSCs proliferative properties had no significant differences. (E) Desmin/Ki67 or mGFP/Ki67 double staining in serial sections demonstrated that Desmin^+^ HSCs proliferative properties significantly increased in BDL‐induced fibrotic liver, but mGFP^+^ HSCs proliferative properties had no significant differences. Arrows depict BrdU^+^ or Ki67^+^ HSCs. *n* = 5 per group. Data are reported as means ± SEM. Comparisons between two groups were performed using the unpaired two‐tailed Student's *t*‐test. Statistical significance was presented at the level of *p* > 0.05 (ns), *p < 0.05, **p < 0.01, ***p < 0.001. Scale bar represents 20 μm.

The migration, activation and proliferation of HSCs are an accompanying process.^14^, ^42^ So, we want to know the proliferative capacity difference between mGFP^+^ and Desmin^+^ HSCs. Immunohistochemical staining of Desmin and mGFP showed that the number of Desmin^+^ HSCs increased remarkably in BDL‐induced fibrotic livers (Figure 3A), but the number of mGFP^+^ HSCs were comparable (Figure 3B). And mGFP/Desmin co‐staining showed that the percentage of mGFP^+^ HSCs accounted for Desmin^+^ HSCs was 49.83% in sham‐operated livers but decreased to 23.84% in BDL‐induced fibrotic livers (Figure 3C), which further confirmed that the number of Desmin^+^ HSCs increased remarkably but the number of mGFP^+^ HSCs had no significant differences in BDL‐induced fibrotic livers. And BrdU labelling showed that 4.74% Desmin^+^ HSCs and 4.34% mGFP^+^ HSCs were BrdU^+^ in sham‐operated livers, but 9.58% Desmin^+^ HSCs and 3.31% mGFP^+^ HSCs were BrdU^+^ in BDL‐induced livers (Figure 3D). Immunostaining of proliferation marker protein Ki‐67 (Ki67) got a similar result, which showed that Desmin^+^ HSCs proliferated significantly in BDL‐induced fibrotic livers (6.57% Desmin^+^ HSCs are Ki67^+^ in sham‐operated livers and 10.59% Desmin^+^ HSCs are Ki67^+^ in BDL‐induced fibrotic livers), but mGFP^+^ HSCs had no notable differences (5.84% mGFP^+^ HSCs are Ki67^+^ in sham‐operated livers and 5.33% mGFP^+^ HSCs in BDL‐induced fibrotic livers) (Figure 3E). Combined with the above results, we concluded that Desmin^+^ HSCs increased greatly in BDL‐induced fibrotic livers, but mGFP^+^ HSCs were not remarkably different. We verified whether ReelinCreERT2‐mediated mGFP expression corresponded to endogenous Reelin expression by conducting mGFP/Reelin double staining, which revealed that ReelinCreERT2‐mediated mGFP expression closely matched endogenous Reelin (Figure S2A). To further investigate the expression of Reelin and Desmin in the liver, we examined wild‐type (WT) and Reelin^CreERT2^; R26T/G^f^ mouse livers. Our data showed that after BDL operation, Desmin^+^ HSCs proliferated significantly and aggregated in both WT and Reelin^CreERT2^; R26T/Gf mouse livers, whereas Reelin^+^ HSCs neither proliferated nor aggregated in either of these groups (Figure S2B,C). And there were no significant differences in the expression of Reelin and Desmin between sham‐operated and BDL‐operated WT and Reelin^CreERT2^; R26T/G^f^ mouse livers (Figure S2B,C).

### 3.4 ReelinCreERT2‐labelled and Desmin^+^ HSCs accumulated around the central vein and are activated in CCl_4_ ‐induced fibrotic livers

**FIGURE 4:**
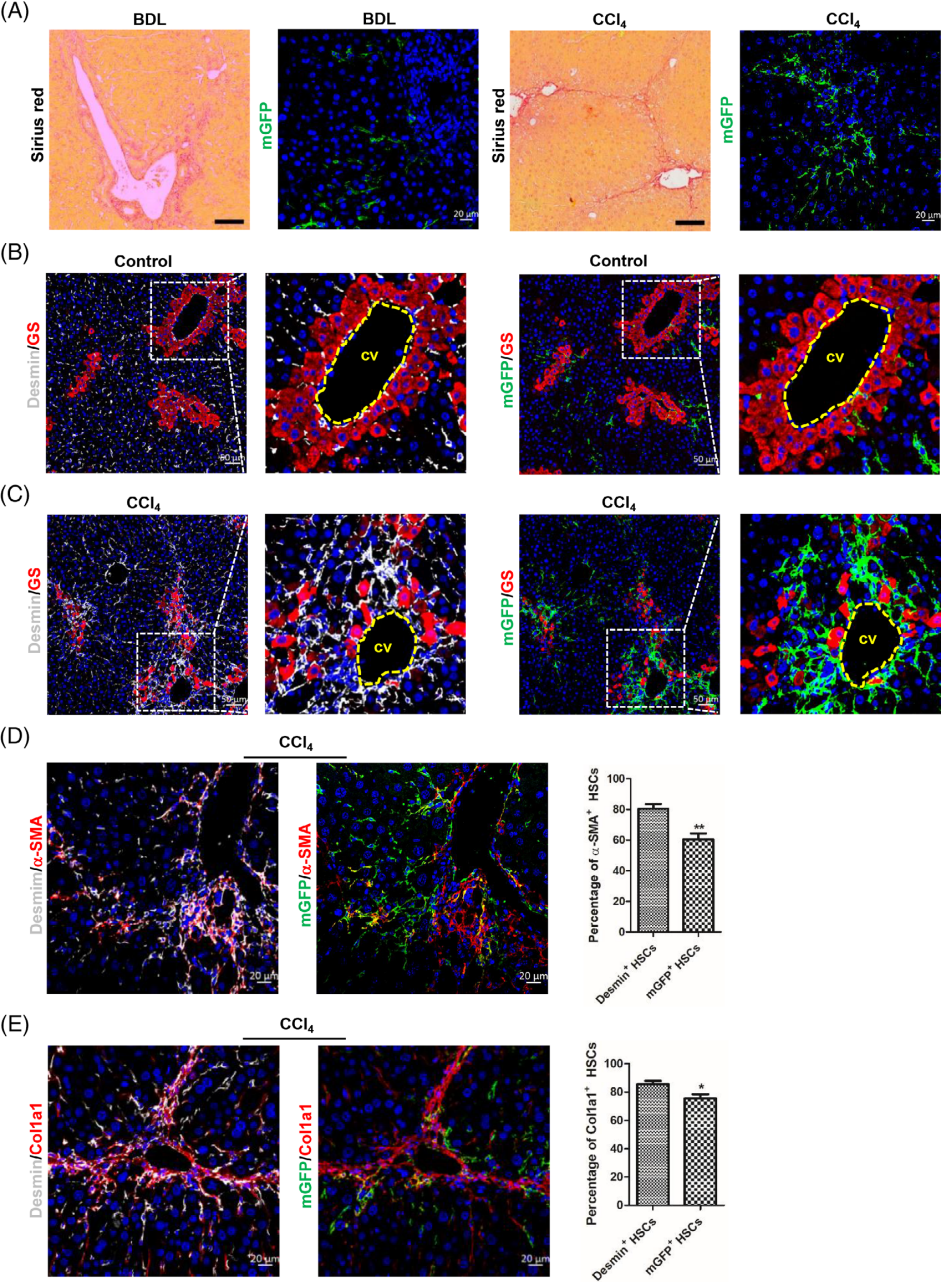
ReelinCreERT2‐labelled hepatic stellate cells (HSCs) accumulated around the central vein and fewer are activated compared to Desmin^+^ HSCs in carbon tetrachloride (CCl_4_)‐induced injured livers. (A) Sirius red and membrane‐tagged green fluorescence protein (mGFP) staining indicated that mGFP expression was different between bile duct ligation (BDL)‐induced biliary fibrosis and CCl_4_‐induced pericentral fibrosis. (B) Desmin/glutamine synthetase (GS) or mGFP/GS double staining in serial sections showed that mGFP^+^ HSCs and Desmin^+^ HSCs were scattered throughout the parenchyma in normal livers. (C) Desmin/GS or mGFP/GS double staining in serial sections showed that both mGFP^+^ HSCs and Desmin^+^ HSCs accumulated around the central vein (cv) and fibrous septa. (D) Desmin/α‐SMA or mGFP/α‐SMA double staining observed that mGFP^+^ HSCs were activated and fewer mGFP^+^ HSCs expressed α‐SMA in CCl_4_‐induced fibrotic livers. (E) Desmin/Col1a1 or mGFP/Col1a1 double staining observed that mGFP^+^ HSCs were activated and fewer mGFP^+^ HSCs expressed Col1a1 in CCl_4_‐induced fibrotic livers; *n* = 5 per group. Data are reported as means ± SEM. Comparisons between two groups were performed using the unpaired two‐tailed Student's *t*‐test. Statistical significance was presented at the level of *p* > 0.05 (ns), *p < 0.05, **p < 0.01, ***p < 0.001. Scale bar in Sirius red staining represents 100 μm and in mGFP immunostaining represents 20 μm in A. Scale bar in B and C represent 50 μm. Scale bar in D and E represents 20 μm.

BDL initially induced biliary duct hyperplasia and further caused biliary fibrosis, however, CCl_4_‐induced liver fibrosis started on pericentral cell injury and formed fibrous septum (Figure 4A). And mGFP^+^ HSCs were scattered throughout the parenchyma in BDL‐caused biliary fibrosis but gathered in CCl_4_‐induced pericentral fibrosis (Figure 4A). So, we wanted to know whether there were differences in migration, activation and proliferation between mGFP^+^ and Desmin^+^ HSCs in CCl_4_‐induced liver injury. After treated with CCl_4_, obvious collagen fibre was observed by Sirius red staining in mouse livers (Figure S3A), and immunostaining showed that the expression of α‐SMA and Col1a1 was significantly increased (Figure S3B). Immunohistochemistry for mGFP/Desmin co‐staining showed that both mGFP^+^ and Desmin^+^ HSCs gathered in CCl_4_‐induced fibrotic livers (Figure S4A). And we explored characteristics of mGFP^+^ and Desmin^+^ HSCs in migration in CCl_4_‐induced injured livers. We observed that both mGFP^+^ and Desmin^+^ HSCs were scattered throughout the parenchyma in healthy livers (Figure 4B) and accumulated around the central vein in CCl_4_‐induced fibrotic livers (Figure 4C). These findings indicated that the migration ability of mGFP^+^ HSCs and Desmin^+^ HSCs was similar in CCl_4_‐induced fibrotic livers. Then, we explored the activation property of mGFP^+^ HSCs in CCl_4_‐induced fibrotic livers. We observed that mGFP^+^ HSCs were activated to MFs after Reelin^CreERT2^; R26T/G^f^ mice treated with CCl_4_ (Figure S4B). And in CCl_4_‐induced fibrotic livers, the immunohistochemistry results showed that 60.43% mGFP^+^ HSCs and 80.37% Desmin^+^ HSCs expressed α‐SMA (Figures 4D and S4C), meanwhile, 75.38% mGFP^+^ HSCs and 85.42% Desmin^+^ HSCs expressed Col1a1 (Figures 4E and S4D). These results indicated that most mGFP^+^ HSCs were activated in CCl_4_‐induced fibrotic livers, and there is more activated Desmin^+^ HSCs than mGFP^+^ HSCs.

### 3.5 ReelinCreERT2‐labelled and Desmin^+^ HSCs proliferate significantly in CCl_4_ ‐induced fibrotic livers

**FIGURE 5:**
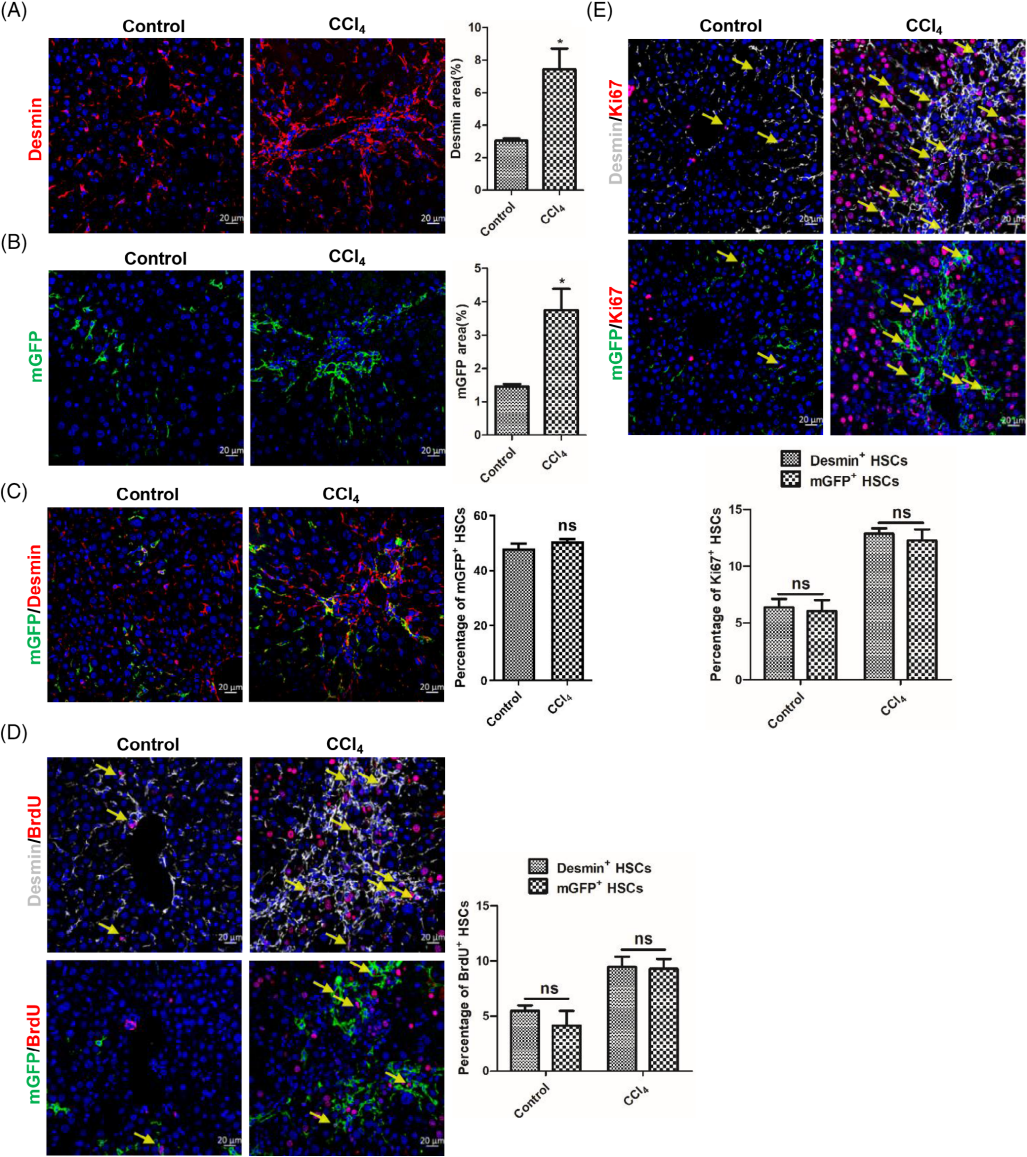
GFP^+^ and Desmin^+^ hepatic stellate cells (HSCs) proliferated in carbon tetrachloride (CCl_4_)‐induced fibrotic livers. (A) Desmin immunostaining displayed that the number of Desmin^+^ HSCs significantly increased in CCl_4_‐induced fibrotic livers. (B) membrane‐tagged green fluorescence protein (mGFP) immunostaining displayed that the number of mGFP^+^ HSCs significantly increased in CCl_4_‐induced fibrotic livers. (C) mGFP/Desmin double staining indicated that the percentage of mGFP^+^ HSCs accounted for Desmin^+^ HSCs had no significant differences in CCl_4_‐induced fibrotic livers. (D) Desmin/bromodeoxyuridine (BrdU) or mGFP/BrdU double staining in serial sections showed that Desmin^+^ HSCs and mGFP^+^ HSCs proliferative properties significantly increased in CCl_4_‐induced fibrotic livers. (E) Desmin/Ki67 or mGFP/Ki67 double staining in serial sections showed that Desmin^+^ HSCs and mGFP^+^ HSCs proliferative properties significantly increased in CCl_4_‐induced fibrotic livers. Arrows depict BrdU^+^ or Ki67^+^ HSCs; *n* = 5 per group. Data are reported as means ± SEM. Comparisons between two groups were performed using the unpaired two‐tailed Student's *t*‐test. Statistical significance was presented at the level of *p* > 0.05 (ns), *p < 0.05, **p < 0.01, ***p < 0.001. Scale bar represents 20 μm.

We also explored the proliferative property of mGFP^+^ HSCs in CCl_4_‐induced fibrotic livers. Immunostaining of Desmin and mGFP showed that the number of Desmin^+^ and mGFP^+^ HSCs increased greatly in CCl_4_‐treated fibrotic livers (Figure 5A, B). And mGFP/Desmin co‐staining revealed that the percentage of mGFP^+^ HSCs accounted for Desmin^+^ HSCs had no significant differences in CCl_4_‐treated fibrotic livers (Figure 5C), which indicated that there were no significant differences between Desmin^+^ and mGFP^+^ HSCs in proliferation. To further confirm the result, we investigated the proliferative property of mGFP^+^ and Desmin^+^ HSCs by BrdU labelling and Ki67 staining in normal and CCl_4_‐treated livers. The result showed that Desmin^+^ HSCs and mGFP^+^ HSCs had superior proliferation ability in CCl_4_‐treated livers compared to those in normal livers, and the proliferation rate was comparable between Desmin^+^ HSCs and mGFP^+^ HSCs (Figure 5D,E). The above results suggested that mGFP^+^ HSCs proliferated significantly, and the proliferation ability of mGFP^+^ HSCs and Desmin^+^ HSCs had no significant differences in CCl_4_‐induced injured livers. In addition, we also investigated Reelin expression in normal and CCl_4_‐induced fibrotic livers in both WT and Reelin^CreERT2^; R26T/G^f^ mice. The results showed cells expressing Reelin and Desmin proliferated significantly and gathered, and there were no significant differences in Reelin and Desmin expression between WT and Reelin^CreERT2^; R26T/G^f^ mice (Figure S5A,B).

### 3.6 ReelinCreERT2‐labelled HSCs do not transdifferentiate into hepatocytes or cholangiocytes in mouse livers

**FIGURE 6:**
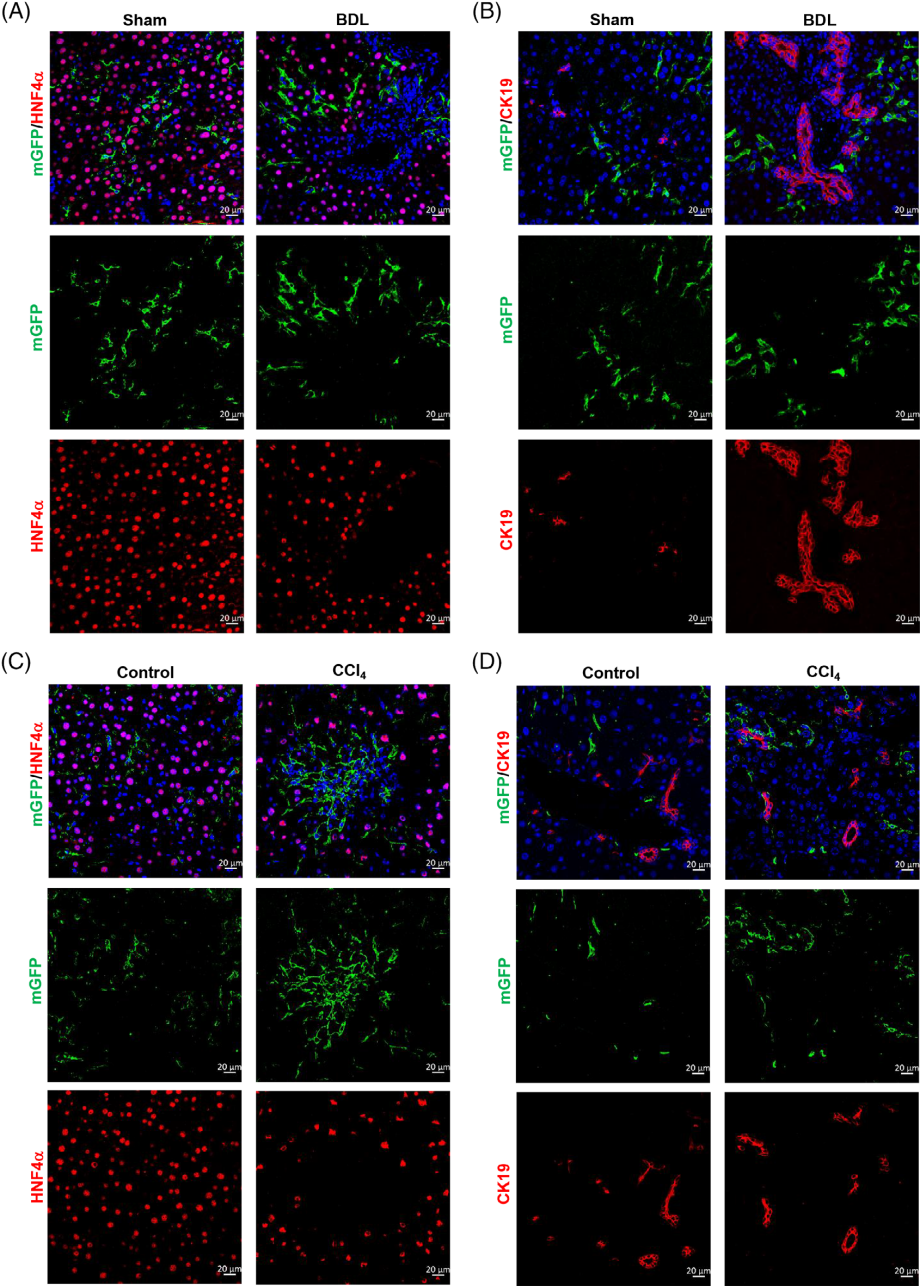
ReelinCreERT2‐labelled hepatic stellate cells (HSCs) do not undergo MET in response to chronic liver injury. (A) membrane‐tagged green fluorescence protein (mGFP^+^) HSCs did not express hepatocytes marker HNF4α in sham‐operated livers or bile duct ligation (BDL)‐induced fibrotic livers. (B) mGFP^+^ HSCs did not express cholangiocytes marker cytokeratin 19 (CK19) in sham‐operated livers or BDL‐induced fibrotic livers. (C) mGFP^+^ HSCs did not express hepatocytes marker HNF4αin livers treated with vehicle or in response to CCl_4_. (D) mGFP^+^ HSCs did not express cholangiocytes marker CK19 in livers treated with vehicle or in response to CCl_4_. Scale bar represents 20 μm.

HSCs transdifferentiating into hepatocytes or cholangiocytes through MET in injured livers is controversial.^29^, ^43^, ^44^ To verify whether HSCs transdifferentiate into hepatocytes and cholangiocytes, we investigated ReelinCreERT2‐labelled HSCs transformation in sham‐operated and BDL‐induced livers. However, analysed immunostaining of mGFP and hepatocyte nuclear factor 4 alpha (HNF4α) showed no mGFP^+^ HSCs expressed HNF4α either in sham‐operated livers or BDL‐induced fibrotic livers (Figure 6A). Moreover, mGFP and cytokeratin 19 (CK19) immunostaining showed no mGFP^+^ HSCs expressed CK19 either (Figure 6B). We also investigated whether ReelinCreERT2‐labelled HSCs transdifferentiate into hepatocytes or cholangiocytes through MET in CCl_4_‐induced fibrotic livers. mGFP/HNF4α and mGFP/CK19 immunostaining in normal and CCl_4_‐treated livers showed no mGFP^+^ HSCs expressed HNF4α or CK19 either (Figure 6C,D). Collectively, these findings excluded the possibility that ReelinCreERT2‐marked HSCs transdifferentiated into hepatocytes or cholangiocytes through MET in healthy or injured mouse livers.

### 3.7 Desmin^+^ HSCs and Vimentin^+^ HSCs have common features

**FIGURE 7:**
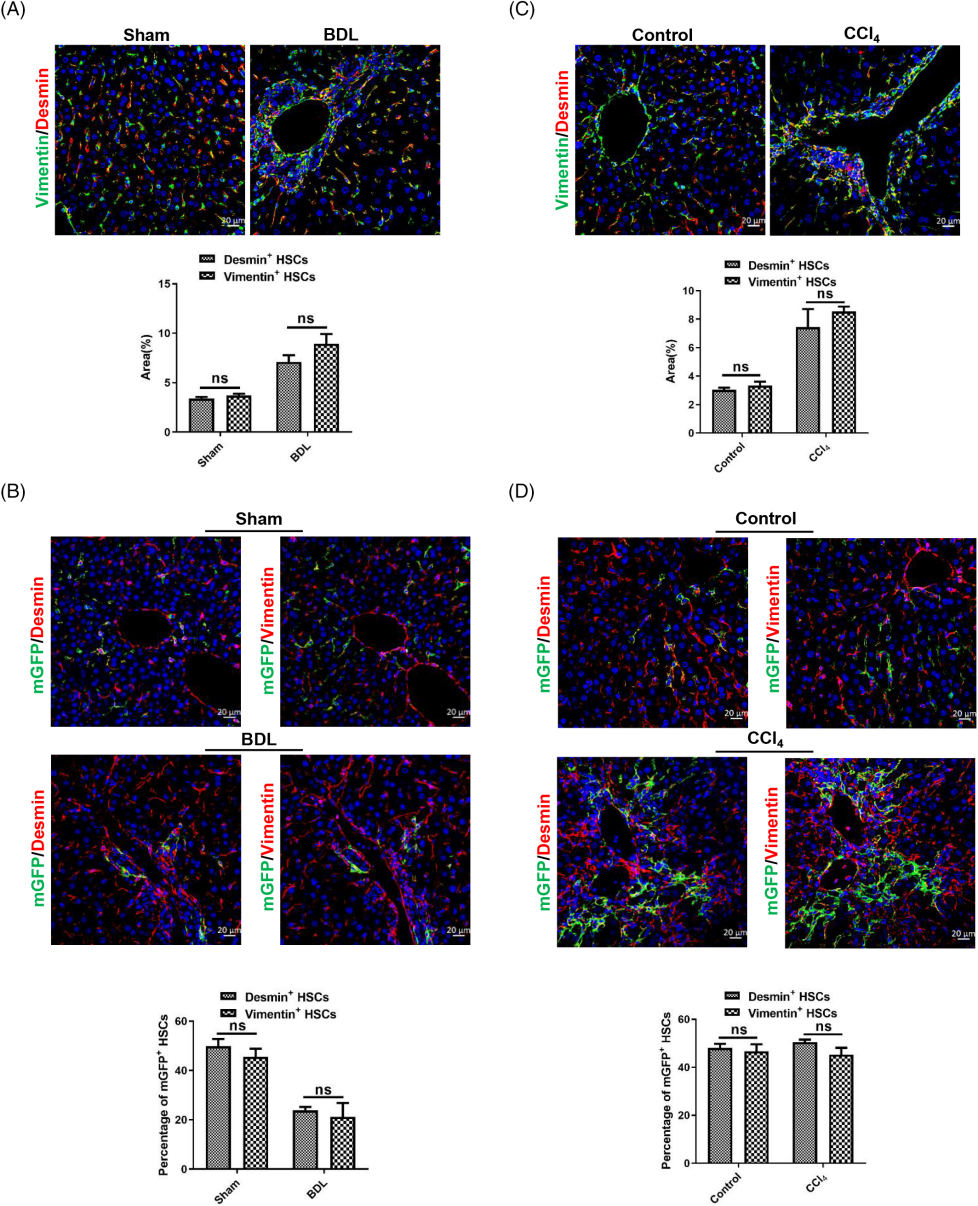
The characteristics of Vimentin^+^ hepatic stellate cells (HSCs) are similar to those of Desmin^+^ HSCs in mouse livers. (A) Vimentin/Desmin double staining indicated that the number and distribution of Vimentin^+^ HSCs were consistent with Desmin^+^ HSCs in sham‐operated and bile duct ligation (BDL)‐operated mouse livers. (B) Desmin/mGFP or Vimentin/mGFP double staining in serial sections indicated that the percentage of mGFP^+^ HSCs accounted for Desmin^+^ HSCs was similar to that accounted for Vimentin^+^ HSCs in sham‐operated and BDL‐operated mouse livers. **(**C) Vimentin/Desmin double staining indicated that the number and distribution of Vimentin^+^ HSCs were consistent with Desmin^+^ HSCs in normal and carbon tetrachloride (CCl_4_)‐treated mouse livers. (D) Desmin/mGFP or Vimentin/mGFP double staining in serial sections indicated that the percentage of mGFP^+^ HSCs accounted for Desmin^+^ HSCs was similar to that accounted for Vimentin^+^ HSCs in normal and CCl_4_‐treated mouse livers; *n* = 5 per group. Data are reported as means ± SEM. Comparisons between two groups were performed using the unpaired two‐tailed Student's *t*‐test. Statistical significance was presented at the level of *p* > 0.05 (ns), *p < 0.05, **p < 0.01, ***p < 0.001. Scale bar represents 20 μm.

Our research indicated that ReelinCreERT2‐labelled HSCs displayed different properties from total HSCs in cholestatic liver injury model but shared similar properties to total HSCs in hepatotoxic liver injury model. To confirm these findings, we chose Vimentin as another total HSC marker.^20^ Desmin and Vimentin immunostaining showed that Vimentin^+^ HSCs characteristics were similar to Desmin^+^ HSCs in sham‐operated and BDL‐induced livers (Figure 7A). mGFP/Desmin and mGFP/Vimentin double immunostaining showed that the percentage of mGFP accounted for Vimentin^+^ HSCs had no differences compared to that mGFP accounted for Desmin^+^ HSCs in sham‐operated and BDL‐induced livers (Figure 7B). And the results of immunostaining of mGFP, Desmin and Vimentin in normal and CCl_4_‐treated livers were consistent with results in sham‐operated and BDL‐induced livers (Figure 7C,D). In addition, HSC activation is associated with inflammation and fibrogenesis, and fibrogenesis is positively correlated with increase in markers of macrophages. Therefore, we investigated the effect of macrophages on mGFP^+^ and Desmin^+^ HSCs. CD68/mGFP and CD68/Desmin co‐staining demonstrated that CD68^+^ macrophages significantly increased and gathered in both BDL‐ and CCl_4_‐treated livers, which was consistent with Desmin^+^ HSCs (Figure S6A, B). Our findings suggest that macrophages may have a greater effect on Desmin^+^ HSCs, although further research is needed to fully understand the effect of macrophages on both mGFP^+^ and Desmin^+^ HSCs.

## 4 DISCUSSION

Reelin as a serine protease is associated with liver fibrosis.^3^, ^45^ But the type of cells expressing Reelin in livers is controversial. Cell lineage tracing is a technique to track cell fate based on cre‐lox system.^25^ Early studies applied cell lineage tracking to investigate stem cells, epithelial‐to‐mesenchymal transition (EMT) and MET.^43^ In this study, we changed the conventional usage of cell lineage tracking and demonstrate that ReelinCreERT2‐labled cells are HSCs. Moreover, these ReelinCreERT2‐labled HSCs (Reelin^+^ HSCs) were a new subset, which displayed differences from Desmin^+^ HSCs in cholestatic liver and similarities to Desmin^+^ HSCs in hepatotoxic liver. Our results showed that Desmin^+^ HSCs accumulated around the portal vein with significant proliferation and activation, whereas Reelin^+^ HSCs did not accumulate either proliferate, and only a small part was activated in BDL‐induced fibrotic livers. However, in CCl_4_‐induced fibrotic livers, both Desmin^+^ HSCs and Reelin^+^ HSCs accumulated around the central vein with remarkable activation activity. Besides, the proliferation potential analysis between Desmin^+^ HSCs and Reelin^+^ HSCs showed Desmin^+^ HSCs and Reelin^+^ HSCs both proliferate markedly in CCl_4_‐induced fibrotic livers (Table 1).

**TABLE 1:** Characteristics of Reelin^+^ HSCs and Desmin^+^ HSCs.

HSCs, %	Normal		BDL		CCl_4_	
	Reelin^+^	Desmin^+^	Reelin^+^	Desmin^+^	Reelin^+^	Desmin^+^
Area	1.53 ± 0.05	3.21 ± 0.12	1.61 ± 0.13	7.10 ± 0.69	3.75 ± 0.64	7.43 ± 1.28
α‐SMA^+^	NA	NA	31.01 ± 4.35	72.35 ± 3.00	60.83 ± 4.1	80.47 ± 3.41
Col1a1^+^	NA	NA	48.53 ± 4.41	89.34 ± 3.28	75.38 ± 3.02	85.42 ± 2.42
BrdU^+^	4.24 ± 0.70	5.11 ± 0.32	3.31 ± 1.43	9.58 ± 0.77	9.28 ± 0.90	9.46 ± 0.91
Ki67^+^	5.95 ± 0.58	6.47 ± 0.37	5.33 ± 0.63	10.59 ± 0.75	12.29 ± 0.96	12.87 ± 0.49
HNF4α^+^	0	NA	0	NA	0	NA
CK19^+^	0	NA	0	NA	0	NA

*Note*: Reelin^CreERT2^, R26T/G^f^ mouse model was used to trace HSCs expressing Reelin and their progeny. And characteristics of Reelin^+^ HSCs and Desmin^+^ HSCs in normal livers (sham‐operated and control livers), BDL‐operated livers and CCl_4_‐treated livers were summarized.

**Abbreviations:** BDL, bile duct ligation; BrdU^+^, bromodeoxyuridine; CCl_4_, carbon tetrachloride; CK19^+^, cytokeratin 19; HNF4α^+^, hepatocyte nuclear factor 4 alpha; HSC, hepatic stellate cell; NA, not applicable.

Single‐cell RNA sequencing is a powerful method to reveal gene expression differences between cells and has divided HSCs into different clusters in normal or injured livers based on gene expression profile,^13^, ^46^ whereas it has failed to select a specific molecule to distinguish an HSC cluster which owns unique properties. So, it is hard to cure liver injury by targeted therapy. Besides, physiological conditions are different from in vitro culture environments, for instance, cellular microenvironment, cytokines, cell–cell junction, etc, which might change the gene expression and chromatin state of HSCs,^17^, ^47^ and so single‐cell RNA might get an incorrect result. Compared to single‐cell RNA sequencing, our cell lineage tracking findings are closer to Reelin^+^ HSCs physiological properties in vivo. And to verify HSCs transforming into hepatocytes and cholangiocytes through MET, we traced Reelin^+^ HSC fate both in BDL‐ and CCl_4_‐induced injured livers and we observed no Reelin^+^ HSCs expressed HNF4α or CK19 (Table 1). In consideration of ReelinCreERT2 only marked part of HSCs, it is still possible that HSCs which were not marked by ReelinCreERT2 differentiate into cholangiocytes or hepatocytes through MET. Whether HSCs differentiate into cholangiocytes or hepatocytes through MET should be further investigated.

In our study, we have not elucidated why there are marked differences in migration, activation and proliferation between Reelin^+^ HSCs and Desmin^+^ HSCs. In consideration of the heterogeneity of HSCs,^46^, ^48^ maybe the gene expression profiling between Reelin^+^ HSCs and Desmin^+^ HSCs is different. Macrophages have been identified as key regulators of liver inflammation, playing a critical role in the progression or resolution of liver fibrosis. Upon activation, macrophages release various cytokines that can cause direct damage to liver parenchymal cells, enhance inflammatory cell infiltration and activate HSCs. In our study, we observed consistent CD68 expression with Desmin in BDL‐ and CCl4‐induced liver injuries, suggesting that macrophages may have a greater effect on Desmin^+^ HSCs. In addition, the mechanisms of fibrogenesis are different in BDL‐ and CCl_4_‐induced liver injuries.^31^ The differences in migration, activation and proliferation between Reelin^+^ HSCs and Desmin^+^ HSCs maybe also caused by the model‐specific influence. The mechanisms of fibrogenesis in BDL‐ and CCl_4_‐induced liver injuries have some important differences. In the BDL‐induced liver injury model, cholestasis‐induced liver injury is the primary mechanism of fibrogenesis, leading to cholangiocyte injury, inflammation, HSC activation, and the deposition of extracellular matrix proteins such as collagen within the liver parenchyma.^21^, ^49^ Toxic bile acids build up in the liver, which can activate HSCs and promote their transformation into MFs. These MFs migrate to areas of liver injury and deposit collagen, resulting in fibrosis.^32^, ^50^ In contrast, CCl_4_ administration induces liver injury through direct hepatotoxicity followed by an inflammatory response that enhances fibrogenesis.^51^, ^52^ Acute liver injury and necrosis occur due to CCl_4_ administration, leading to Kupffer cell activation and the release of pro‐inflammatory cytokines such as tumour necrosis factor‐alpha (TNF‐α), interleukin‐1β (IL‐1β), and interleukin‐6 (IL‐6).^32^, ^53^ This inflammatory environment promotes the activation of HSCs into MFs, leading to fibrosis.^54^ It is possible that hepatotoxicity has a greater effect on HSCs than cholestasis. Future studies should combine metabolomics, transcriptomics and lineage tracking to identify the mechanisms which contribute to the differences in migration, activation and proliferation between Reelin^+^ HSCs and Desmin^+^ HSCs in cholestatic and hepatotoxic liver.

In conclusion, we using cell lineage tracking demonstrated that ReelinCreERT2‐labled HSCs are a new subset, which display different properties in BDL‐induced fibrotic livers and similar characteristics in CCl_4_‐induced fibrotic livers compared to Desmin^+^ HSCs. And we observed no ReelinCreERT2‐labled HSCs underwent MET. Our findings enlighten that treating liver fibrosis caused by different etiologies should suit the remedy to the case. For instance, focusing on Reelin^+^ HSCs may be a good therapy target in hepatotoxic liver, however, target the Reelin^−^ HSCs may optimize the therapeutic effects in cholestatic liver.

## AUTHOR CONTRIBUTIONS

Ning Chen and Lisheng Zhang conceived and designed the study; Ning Chen provided the experimental data; Ning Chen and Shenghui Liu performed the experiments; Dan Qin, Dian Guan, Yaqing Chen, Chenjiao Hou, and Songyun Zheng helped in animal experiments; Ning Chen and Lisheng Zhang discussed and drafted the manuscript; Liqiang Wang and Xiangmei Chen reviewed the manuscript; Wei Chen provided important suggestions and assistance throughout the entire process of revising the manuscript; Lisheng Zhang and Ning Chen organized the data and wrote the manuscript.

## FUNDING INFORMATION

This work was supported by National Key R&D Plan no. 2017YFA0103202 and no. 2017YFA0103200, National Natural Science Foundation of China 32071143, the Fundamental Research Funds for the Central Universities (2662019YJ008), and Huazhong Agricultural University Startup funds.

## CONFLICT OF INTEREST STATEMENT

The authors declare no competing interests.

## Supporting information


**Data S1:** Supporting Information

## Data Availability

The data that support the findings of this study are available from the corresponding author upon reasonable request.
