# Supplementary material for: Fate tracking reveals differences between Reelin+ hepatic stellate cells (HSCs) and Desmin+ HSCs in activation, migration and proliferation
Source: Cell Prolif. 2023 May 28;56(12):e13500. doi: 10.1111/cpr.13500 (PMC10693182; doi:10.1111/cpr.13500)
Supplement: Supplementary file 1 — Data S1: Supporting Information [file CPR-56-e13500-s001.docx]

**Supplemental information**





**Figure S1. Reelin is not expressed in hepatocytes or hepatoblasts and BDL induces** **severe liver fibrosis.**

**A.** mGFP/HNF4α or mGFP/EpCAM double staining indicated that hepatocytes or hepatoblasts did not express mGFP. **B.** Sirius red staining displayed that severe biliary fibrosis was induced by BDL**. C.** α-SMA and Col1a1 immunostaining indicated that α-SMA and Col1a1 expression were significantly increased in BDL-operated Reelin^CreERT2^; R26T/G^f^ mice**.** n=5 per group. Data are reported as means ± SEM. Comparisons between two groups were performed using the unpaired two-tailed Student’s t-test. Statistical significance was presented at the level of p > 0.05 (ns), *p < 0.05, **p < 0.01, ***p < 0.001. Scale bar in A and C represents 20 μm. Scale bar in B represents 100 μm.


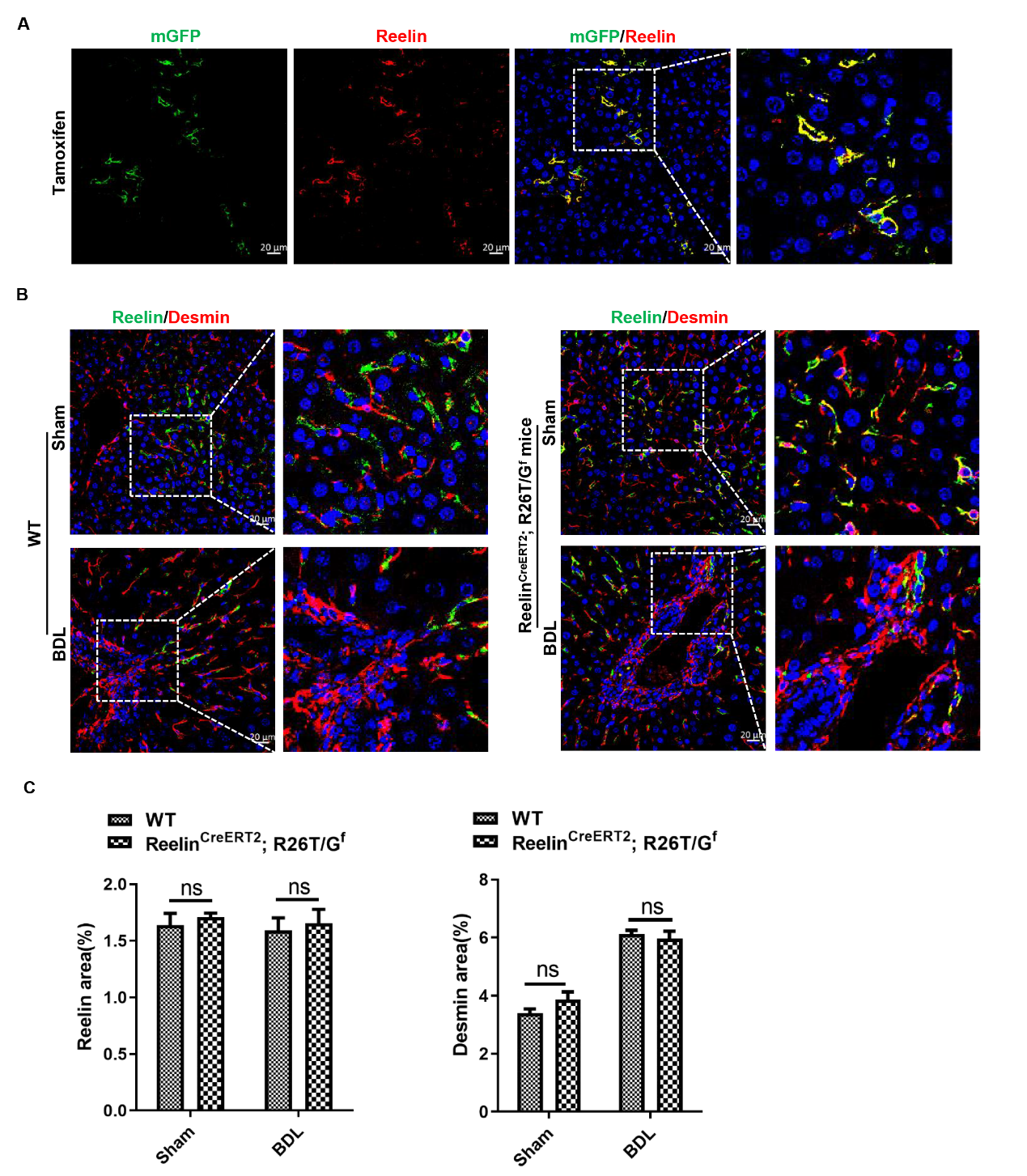


**Figure S2. The expression of****Reelin in** **sham-operated and BDL-operated WT** **and Reelin^CreERT2^; R26T/G^f^ mousedon’t show any significant differences.**

**A.** mGFP/Reelin double staining in untreated WT mouse livers and Reelin^CreERT2^; R26T/G^f^ mouse livers treated with TAM showed overlap between mGFP and Reelin. **B.** Reelin/Desmin double staining showed the expression of Reelin and Desmin in sham-operated and BDL-operated wild-type mouse livers is not different from that in sham-operated and BDL-operated Reelin^CreERT2^; R26T/G^f^ mouse livers treated with TAM. **C.** Quantification of cells expressing Reelin and Desmin in sham-operated and BDL-operated wild-type mouse livers and Reelin^CreERT2^; R26T/G^f^ mouse livers in sham-operated and BDL-operated Reelin^CreERT2^; R26T/G^f^ mouse liver treated with TAM. WT mice, n=3 per group. Reelin^CreERT2^; R26T/G^f^ mice, n=5 per group. Data are reported as means ± SEM. Comparisons between two groups were performed using the unpaired two-tailed Student’s t-test. Statistical significance was presented at the level of p > 0.05 (ns), *p < 0.05, **p < 0.01, ***p < 0.001. Scale bar represents 20 μm.


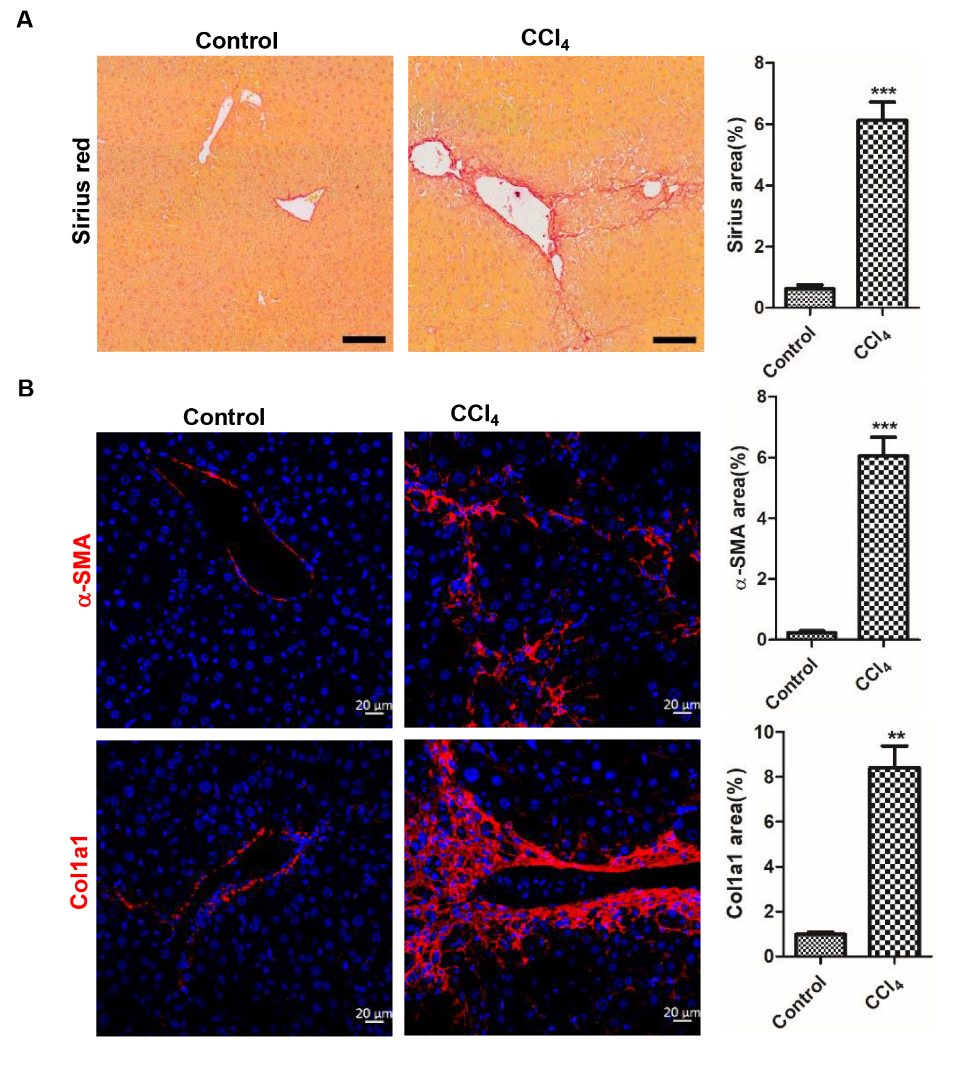


**Figure S3. CCl_4_ induces severe liver fibrosis.**

**A.** Sirius red staining indicated severe pericentral fibrosis was induced by CCl_4_**. B.** α-SMA and Col1a1 Immunostaining indicated that α-SMA and Col1a1 expression were significantly increased in CCl_4_-treated mice**.** n=5 per group. Data are reported as means ± SEM. Comparisons between two groups were performed using the unpaired two-tailed Student’s t-test. Statistical significance was presented at the level of p > 0.05 (ns), *p < 0.05, **p < 0.01, ***p < 0.001. Scale bar in A represents 100 μm. Scale bar in B represents 20 μm.


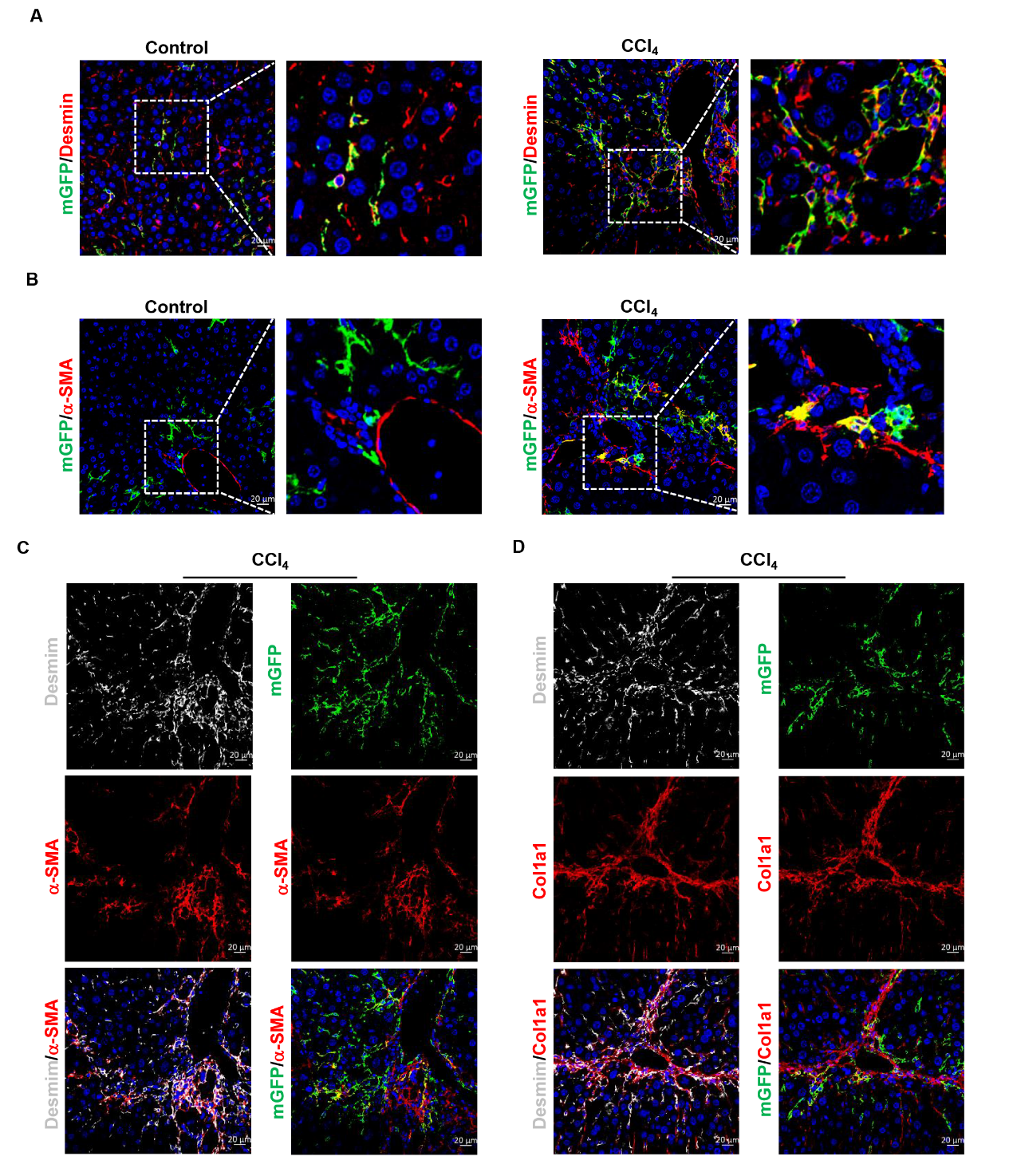


**Figure S4. Reelin^+^** **HSCs accumulate around the vein and are activated in CCl_4_-induced fibrotic livers.**

**A.** Immunohistochemistry of normal or fibrotic livers induced by CCl_4_ showed that mGFP^+^ HSCs gathered in CCl_4_-induced fibrotic livers. **B.** Immunostaining of mGFP and α-SMA showed that mGFP^+^ HSCs were activated to MFs in CCl_4_-induced fibrotic livers. **C.** Separate images of Desmin/α-SMA or mGFP/α-SMA double staining in CCl_4_-induced fibrotic livers. **D.** Separate images of Desmin/Col1a1 or mGFP/Col1a1 double staining in CCl_4_-induced fibrotic livers.Scale bar represents 20 μm.


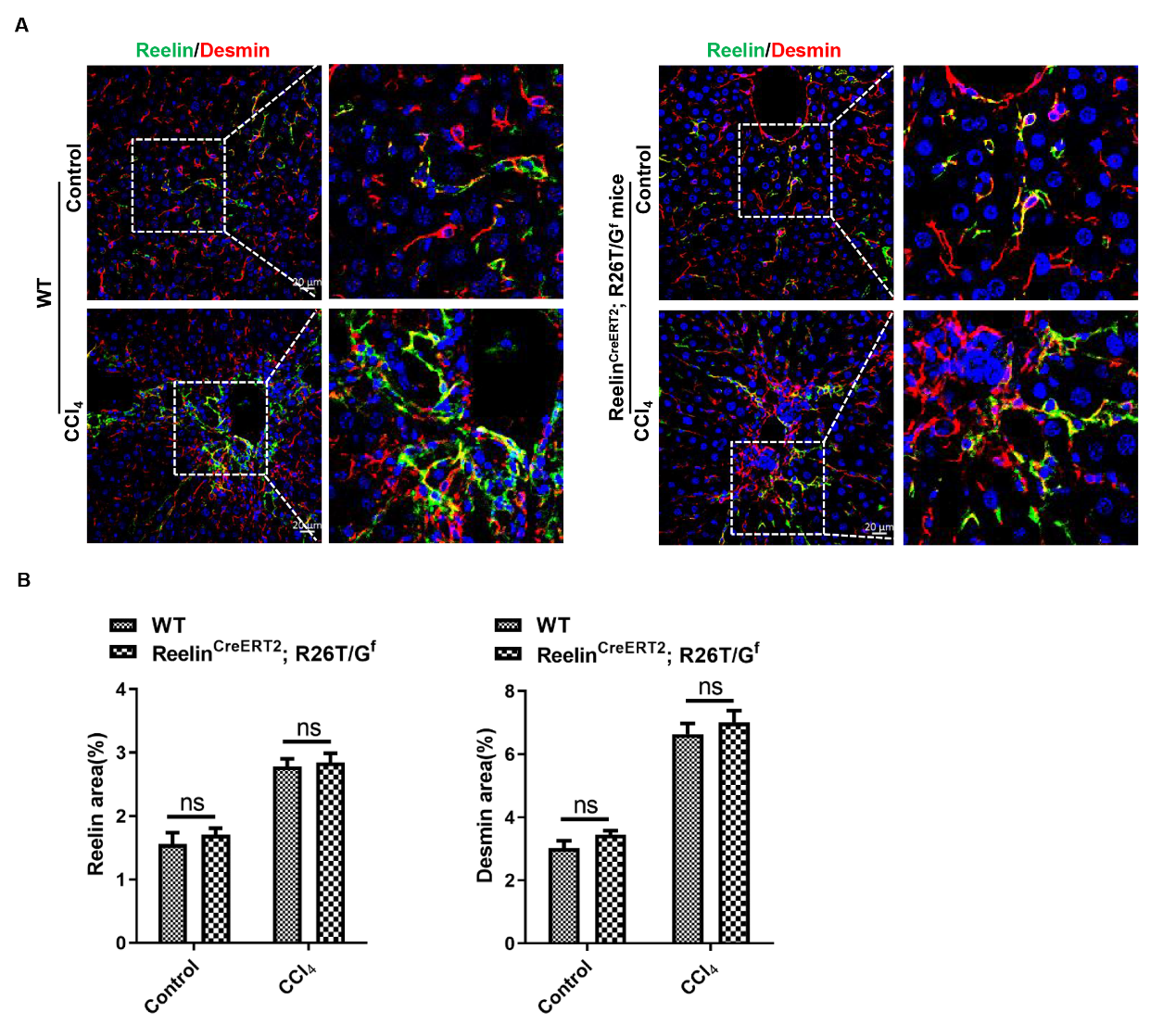


**Figure S5. The expression of Reelin in the livers of control and CCl_4_-treated WT and Reelin^CreERT2^; R26T/G^f^ mouse don’t show any significant differences.**

**A.** Reelin/Desmin double staining showed the expression of Reelin and Desmin in control and CCl_4_-treated wild-type mouse livers was not different from that in control and CCl_4_-treated Reelin^CreERT2^; R26T/G^f^ mouse liver treated with TAM. **B.** Quantification of cells expressing Reelin and Desmin in control and CCl_4_-treated WT mouse livers and in control and CCl_4_-treated Reelin^CreERT2^; R26T/G^f^ mouse liver treated with TAM. WT mice, n=3 per group. Reelin^CreERT2^; R26T/G^f^ mice, n=5 per group. Data are reported as means ± SEM. Comparisons between two groups were performed using the unpaired two-tailed Student’s t-test. Statistical significance was presented at the level of p > 0.05 (ns), *p < 0.05, **p < 0.01, ***p < 0.001. Scale bar represents 20 μm.


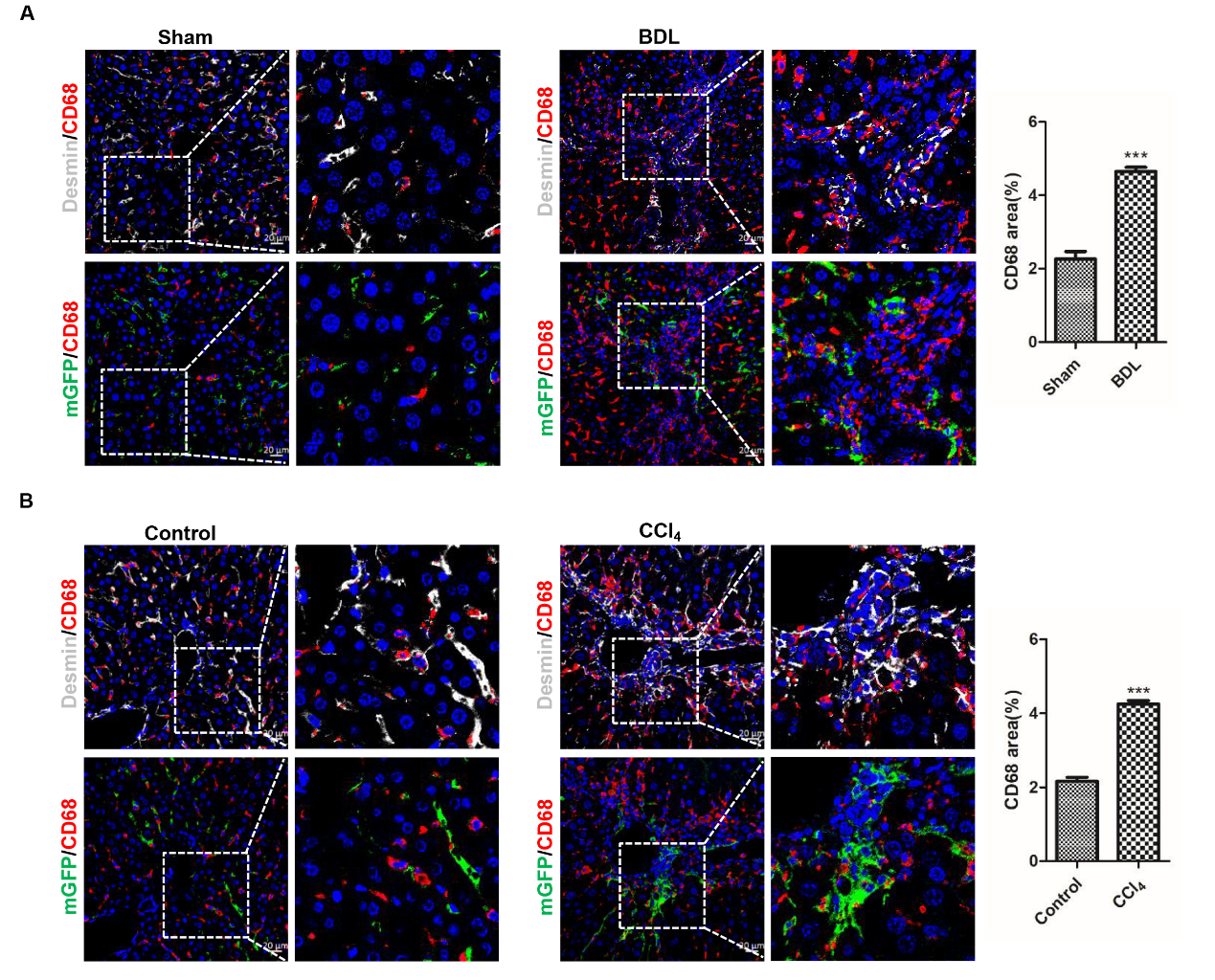


**Figure S6.** **Number of CD68^+^ macrophages is increased and accumulated in BDL-operated and CCl_4_-treated mouse livers which is consistent with Desmin^+^ HSCs.**

**A.** Desmin/CD68 or mGFP/CD68 double staining showed CD68^+^ macrophages increased and gathered in BDL-operated livers consistent with Desmin^+^ HSCs. **B.** Desmin/CD68 or mGFP/CD68 double staining showed CD68^+^ macrophages increased and gathered in CCl_4_-treated livers consistent with Desmin^+^ HSCs. n=5 per group. Data are reported as means ± SEM. Comparisons between two groups were performed using the unpaired two-tailed Student’s t-test. Statistical significance was presented at the level of p > 0.05 (ns), *p < 0.05, **p < 0.01, ***p < 0.001. Scale bar represents 20 μm.
